# Experimental and Natural Warming Elevates Mercury Concentrations in Estuarine Fish

**DOI:** 10.1371/journal.pone.0058401

**Published:** 2013-03-12

**Authors:** Jennifer A. Dijkstra, Kate L. Buckman, Darren Ward, David W. Evans, Michele Dionne, Celia Y. Chen

**Affiliations:** 1 Wells National Estuarine Research Reserve, Wells, Maine, United States of America; 2 Department of Biological Sciences, Dartmouth College, Hanover, New Hampshire, United States of America; 3 Fisheries Biology Department, Humboldt State University, Arcata, California, United States of America; 4 Center for Coastal Fisheries and Habitat Research, National Oceanic and Atmospheric Administration, Beaufort, North Carolina, United States of America; National University of Singapore, Singapore

## Abstract

Marine food webs are the most important link between the global contaminant, methylmercury (MeHg), and human exposure through consumption of seafood. Warming temperatures may increase human exposure to MeHg, a potent neurotoxin, by increasing MeHg production as well as bioaccumulation and trophic transfer through marine food webs. Studies of the effects of temperature on MeHg bioaccumulation are rare and no study has specifically related temperature to MeHg fate by linking laboratory experiments with natural field manipulations in coastal ecosystems. We performed laboratory and field experiments on MeHg accumulation under varying temperature regimes using the killifish, *Fundulus heteroclitus*. Temperature treatments were established in salt pools on a coastal salt marsh using a natural temperature gradient where killifish fed on natural food sources. Temperatures were manipulated across a wider range in laboratory experiments with killifish exposed to MeHg enriched food. In both laboratory microcosms and field mesocosms, MeHg concentrations in killifish significantly increased at elevated temperatures. Moreover, in field experiments, other ancillary variables (salinity, MeHg in sediment, etc.) did not relate to MeHg bioaccumulation. Modeling of laboratory experimental results suggested increases in metabolic rate as a driving factor. The elevated temperatures we tested are consistent with predicted trends in climate warming, and indicate that in the absence of confounding factors, warmer sea surface temperatures could result in greater in bioaccumulation of MeHg in fish, and consequently, increased human exposure.

## Introduction

Methylmercury (MeHg) is a potent neurotoxin that readily bioaccumulates and biomagnifies in coastal marine food webs, which are important pathways to human mercury (Hg) exposure [1]. While it is not presently known how climate change will affect the fate of MeHg in marine ecosystems, climate related factors, specifically rising temperatures, could increase the production and bioaccumulation of MeHg at the base of marine food webs. This, in turn, could affect human exposure through consumption of marine fish. Studies of human populations have shown correlations between total mercury (THg) in fish consumed and increased blood pressure, myocardial infarctions and delays in neurobehavioral development in children [2], [3]. In the US, 50 states have instituted fish consumption advisories pertaining to Hg, and coastal advisories exist for all states bordering the Atlantic and the Gulf of Mexico due to the rising concern regarding health risks associated with Hg exposure through consumption of seafood. In this study, we investigated the effect of temperature on MeHg bioaccumulation in killifish, important and ubiquitous estuarine forage fish, in both the laboratory and in the field.

Marine and estuarine systems are subject to sustained multiple stressors that include climate change [4] and atmospheric deposition and chemical inputs of heavy metals from industry and fossil fuel emissions [5], [6]. The effects of climate change are particularly apparent in coastal ecosystems, as they are exposed to climate extremes [4], [7]. These systems are also used extensively by humans and are venues of exposure for a variety of toxic chemicals [6]. Coastal and estuarine sediments are repositories for Hg, receiving both atmospheric and watershed inputs [8], [9]. Due to the biogeochemical conditions that stimulate methylation of inorganic Hg by sulfate-reducing bacteria [10]–[13], estuaries and coastal zones are hot spots for the transformation of Hg into its more toxic form of MeHg. MeHg produced in coastal zones is available to estuarine food webs that are important pathways to human exposure, as the consumption of marine fish and shellfish are the dominant source of MeHg to humans [1], [14]. The production of MeHg in coastal zones may account for much of the bioaccumulation in marine fish [15], along with methylation in the water column [16], [17].

MeHg production and bioaccumulation in aquatic systems is affected by biological and geochemical factors [18]–[20]. MeHg concentrations in fish and shellfish species are influenced by sediment type and biogeochemistry [21], which affect methylation rates and MeHg flux to the water column, and water chemistry. Climate-induced changes in environmental parameters such as dissolved organic carbon, pH, and temperature can directly or indirectly alter organismal MeHg concentrations through altered sediment metal-binding capacity, increased or decreased methylation rates, microbial activity levels, diet (benthic and pelagic community composition), and fish physiology [22]–[29]. While climate warming and its role in altering heavy metal burdens in fish and shellfish has received attention, forecasting the effects of climate warming on MeHg bioaccumulation is difficult due to the multiple processes that influence MeHg production, bioavailability and uptake [28], [30].

The Northeast US is clearly warming with rising air and sea temperatures and a lengthened growing season [31], [32]. In Northeast coastal waters, annual mean temperature has risen by ∼1°C since the 1970's [33] and by 1–2°C during the summer [4], [34]. Predicted increases in water temperature for temperate waters are between 1.5°C and 4.5°C [35], [36], making an understanding of the effect of temperature on contaminant uptake of particular interest. Approaches to predicting the effect of climate warming on heavy metal burdens have focused primarily on decadal correlations between nutrient concentrations, warmer water temperatures and metal concentrations [8], [37]–[40]. While reported studies on the effect of temperature on MeHg bioaccumulation are rare, particularly in marine environments, in aquatic systems, there is generally a positive correlation between warmer water temperatures and accumulation of heavy metals. Laboratory studies relating temperature to Hg and MeHg bioaccumulation in freshwater zooplankton indicate temperature may increase MeHg accumulation [41]. Seasonal field studies suggest increased Hg methylation in sediments at warmer temperatures, which could lead to greater concentrations of bioavailable MeHg [11], [42], [43]. Freshwater arctic fish have shown increasing contaminant burdens despite decreasing atmospheric deposition, with climate and temperature effects hypothesized to be driving factors for the increased exposure [37]. These and other studies suggest that warmer temperatures may lead to greater heavy metal bioaccumulation in marine ecosystems e.g. [37]. Mechanistically, rising water temperatures can lead to increase feeding rates in response to higher metabolic demand [18], [44]–[46]. Higher consumption could result in greater MeHg uptake and accumulation [41]; conversely increased consumption could also result in greater growth, leading to reduced MeHg bioaccumulation through somatic growth dilution [18], [47]. Given that the global climate is warming and that temperature is increasing at a more rapid rate in temperate regions than previously recorded [36], it is critical to evaluate the relationship between temperature and Hg bioaccumulation in temperate coastal systems. No prior studies have linked climate warming to MeHg bioaccumulation using both laboratory and field related studies. This study provides important insight into the fate of MeHg in marine ecosystems under climate warming scenarios.

We coupled field and laboratory studies to investigate the effect of temperature on MeHg accumulation in the killifish, *Fundulus heteroclitus*. Small fish that reside in estuaries are valuable bioindicators for MeHg uptake in local food webs because their low trophic position links them to MeHg production and entry of MeHg into marine food webs [48], [49]. Both their trophic position and their widespread geographic distribution make killifish a good focal species with which to examine effects of climate warming on MeHg in coastal and marine ecosystems. Temperature induced changes in killifish MeHg bioaccumulation in field studies can result from increased MeHg production and subsequent trophic transfer of MeHg through the food web and/or temperature mediated effects on fish growth [18]. In our study, we examined killifish accumulation of MeHg in natural saltmarsh pools spanning a temperature gradient, investigated the effects of temperature alone on MeHg bioaccumulation in laboratory experiments, and evaluated possible mechanisms of temperature effect on MeHg bioaccumulation.

## Materials and Methods

### Field Studies

The study was conducted in the undeveloped marsh-estuarine ecosystem of Little River estuary within the Wells National Estuarine Research Reserve (Wells NERR) in Wells, Maine located along the southern Gulf of Maine coastline. On July 30, 2009 and May 20, 2010, we enclosed natural populations of killifish in six salt marsh pools of similar size and depth in the Little River Estuary in Wells, ME using rigid 1-m high, 3 mm mesh Vexar™ attached to PVC support poles. The lower edges of the Vexar™ were attached to the pool walls below the water surface using 20-cm long landscaping staples, closing off pools so that fish could not leave during higher tide, but allowed movement of smaller organisms (e.g., zooplankton). Fish were collected on October 15, 2009 and September 15, 2010 at the end of the growing season. Because these individuals were enclosed in salt marsh pools, contaminant concentrations measured for their tissues were assumed to be a reflection of their local environment. Salt marsh pools were selected as they provide habitat for fish species and support significant prey fish (e.g. killifish) production [50]. Our preliminary results found that temperatures in pools varied according to their distance away from the marsh seaward edge. This spatial thermal gradient for pool habitats mimics projected increases in summer and fall sea surface temperatures for the region 1.5°C to 4.5°C [35], [36].

We recorded temperature hourly in each pool using a HOBO temperature data logger placed ∼0.3 m below the water's surface. Salinity and dissolved oxygen concentrations were measured every two weeks using a YSI™dataprobe 85. Sediments from the six pools were collected concurrently with the killifish at the end of the study period and were assumed to reflect relative availability of MeHg within the pools. Fish were sampled using seines and were put in an ice bath prior to being euthanized by severing the spinal cord. They were handled with trace metal clean procedures and frozen for stable isotope and MeHg analysis. A sample of the top 2 cm of the sediment from each pool was randomly collected along the edge of the pool with a 6.5 cm diameter coring tube. Each sediment sample was freeze-dried, homogenized and sent to the Dartmouth College Trace Element Core Facility for Hg and MeHg analysis [51]–[53]. The ratio of MeHg to total Hg was used as a proxy for MeHg production. Percent total organic carbon (%TOC) was measured using thermal partitioning at 550°C. Total lengths and weights of each fish were measured and only fish between 20 mm and 40 mm (n = 3 to 5/pool) were analyzed for this study. Year 1, young-of-the-year (YOY) killifish are less than 50 mm in the fall [54]. Therefore, concentrations of MeHg in killifish used for this study represent accumulation of MeHg for the studied growing season only. Fish were placed in trace-metal clean containers and freeze dried, weighed, homogenized and analyzed for total mercury by EPA Method 7473 [55], using a Milestone DMA-80 analyzer. Recoveries of the CRMs DOLT-3 and TORT-2 averaged 101%±11%and 100%±9%, respectively. Spike recoveries averaged 100.3%±3.6%. MeHg was extracted from the same tissue samples with 4.57 N nitric acid. Aliquots of the extract were analyzed by ethylation, gas-chromatographic cold-vapor atomic fluorescence spectroscopy using a Brooks Rand MERX automated MeHg analyzer [53]. Recoveries for MeHg in a series of marine tissue CRMs from the Canadian National Research Council averaged 106%±3%. Average percent MeHg (with standard errors) of total Hg in killifish was 60.9%±8.9 and 46.3%±4.8 for 2009 and 2010, respectively.

To compare feeding behavior of killifish from separate pools, a sub-sample (1–2 grams) of individual freeze-dried 2010 fish material was analyzed for stable isotopic composition (δ^13^C, δ^15^N) using an Elementar Vario Micro Cube elemental analyzer interfaced to an IsoPrime 100 mass spectrometer (precision = ±0.2‰) at the Environmental Protection Agency, Atlantic Ecology Laboratory in Narragansett, Rhode Island [56], [57]. Samples for C and N were run simultaneously through the unit (i.e., carbon and nitrogen was analyzed using the same sample). Samples were randomly run in batches of 80–100 due to instrument capacity. Laboratory standards (blue mussel) were placed in duplicate every 20^th^ sample and at the beginning and end of each run to check and correct for instrument drift.

### Laboratory Studies

Two experiments using killifish collected from the Wells National Estuarine Research Reserve (size ranges 0.603 g–1.398 g live weight in trial 1 and 1.038 g–1.934 g live weight in trial 2) were carried out for 30 days in March and May 2011. Experimental temperatures were 15°C, 21°C and 27°C. Temperatures were chosen to capture the range of temperatures that occurred during the growing season in the field study and were inclusive of predicted increases in average temperature under climate change scenarios. The range of temperatures was broader than the averages of the field mesocosm temperatures because they captured the extremes measured in the field, not the range of mean temperatures across pools. Fish used for laboratory experiments were YOY and collected during the fall of 2010 from a single location in Wells, ME. The fish had been maintained in aerated aquaria at Dartmouth College until their use. Prior to the beginning of Trial 1, three fish from the same cohort of fish used for both trials were analyzed for MeHg concentrations yielding an average concentration of 125±24 ng g^−1^ dry weight. For each trial, individual fish of similar size and healthy appearance were selected and randomly placed in acid-cleaned 1.5 L plastic containers, one fish per container. Six replicate fish per temperature treatment were analyzed for trial 1, and four replicates per treatment for trial 2. Each container was filled with marine water (30 PSU) created with Instant Ocean and randomly assigned to a temperature treatment. Individual fish were weighed (live wet weight) prior to the beginning and at the end of the study. Beakers were placed in temperature-controlled incubators, covered to prevent fish from escaping and to minimize evaporation, provided with aeration, and left to acclimate for one week. During acclimation, temperatures were changed no more than 3°C per day until the desired temperature (15°C, 21°C, or 27°C) was reached. Fish were fed an unrestricted ration of Tetramin flakes daily prior to the beginning of the experiment. Water changes were carried out three times a week. During the experiment, fish were fed once daily 0.2 g pellets composed of fish meal from Hg contaminated fish, potato starch, and vitamins with an average MeHg (±standard deviation) concentration of 97±1.3 µg/kg dry weight. Fish were kept on a light cycle of 16 hrs. on and 8 hrs. off. Fish were observed daily during feeding to ensure that aeration was sufficient and that the fish were healthy and active. Temperatures were checked daily using a thermometer placed inside each incubator. At the end of the 30-day period, fish were reweighed and euthanized via severing the spinal cord, placed in trace-metal clean vials, and freeze dried. Whole dried fish were weighed, homogenized and samples were analyzed by purge and trap GC using a MERX-M automated methyl mercury analyzer (Brooks Rand Labs, Seattle, WA) at the Dartmouth Trace Element Analysis Core Facility [52], [58]. Total Hg was determined as the sum of methyl and inorganic Hg extract. Percent recovery of MeHg from DORM3 (Canadian National Research Council) averaged 94±1.3% for trial 1 and 91±1.3% for trial 2. Duplicate samples had less than 2%RSD. Detection limits were 3ppb for MeHg and 7ppb for inorganic Hg.

### Ethics Statement

All necessary permits were obtained for the laboratory and the field studies. Maine Department of Resources (permit # SL2009-53-00 and SL2010-60-01) and the Dartmouth College Animal Care and Use Program (protocol # 10-04-06) approved field collection, euthanization, and laboratory fish studies.

### Bioenergetics Model

To address concerns that trial 2 fish may have been food limited, resulting in weight loss, and to assess possible mechanisms of temperature effect on bioaccumulation, results of the laboratory experiments were evaluated using a previously developed killifish bioenergetics model (Growth  =  Consumption-Respiration-Egestion-Excretion) [59]. Methyl mercury bioaccumulation modeling is similar to that of Trudel and Rasmussen [60], with *Fundulus*-specific bioenergetics. This enabled us to derive a mechanistic understanding of the effects of temperature on MeHg bioaccumulation in our killifish laboratory study. Though the model is designed for the species *Fundulus parvipinnis* (west coast equivalent of *Fundulus heteroclitus*), many of the parameters used in the model were borrowed from studies of *F. heteroclitus* allowing us to adapt the model for this study.

Consumption and respiration are both functions of temperature, and egestion and excretion are treated as proportional to consumption. Maximum consumption and respiration occur at 27°C and 29°C, respectively [59]. In the model, consumption is treated as proportional (P) to maximum consumption that is an allometric function of fish weight and of temperature. Our P was estimated by fitting our model to our observed growth rates. Methylmercury bioaccumulation is a function of actual consumption, MeHg concentration in food and efficiency of MeHg assimilation and MeHg excretion which is also temperature dependent [60].

### Statistical Analysis

Data were analyzed using JMP 8.0©. A one-way ANOVA was used to assess differences in stable isotope values and wet weights of killifish among pools in the field studies. A standard least squares multiple regression of mean MeHg concentrations in fish<40 mm collected from individual pools in 2009 and 2010 was used to relate variation in MeHg tissue concentrations to mean temperature, salinity, dissolved oxygen, as well as sediment MeHg concentrations and % sediment TOC. A two-way Analysis of Variance (ANOVA) with trial and treatment as interaction terms was used to investigate effects of temperature on bioaccumulation of MeHg and on arcsine percent change in mass of fish in laboratory experiments. Percent change in mass was transformed to conform to the assumptions of ANOVA (e.g., normality and homogeneity). On finding a significant difference, post-hoc Tukey-Kramer tests set to *P* = 0.05 level of significance were used to assess pairwise differences in treatment means. A one-way ANOVA was used to test for differences in initial biomass of killifish exposed to temperature treatments between the two laboratory trials.

## Results

**Figure 1:**
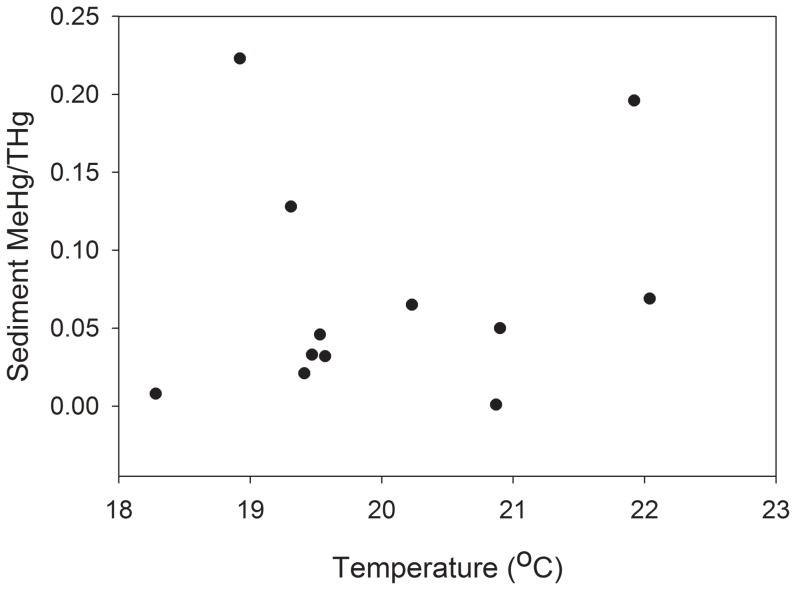
There is no relationship between temperature and %MeHg in sediments for 2009 and 2010 in pool habitats, indicating that temperature may not be affecting MeHg bioavailability and consequently fish exposure between pools.

**Figure 2:**
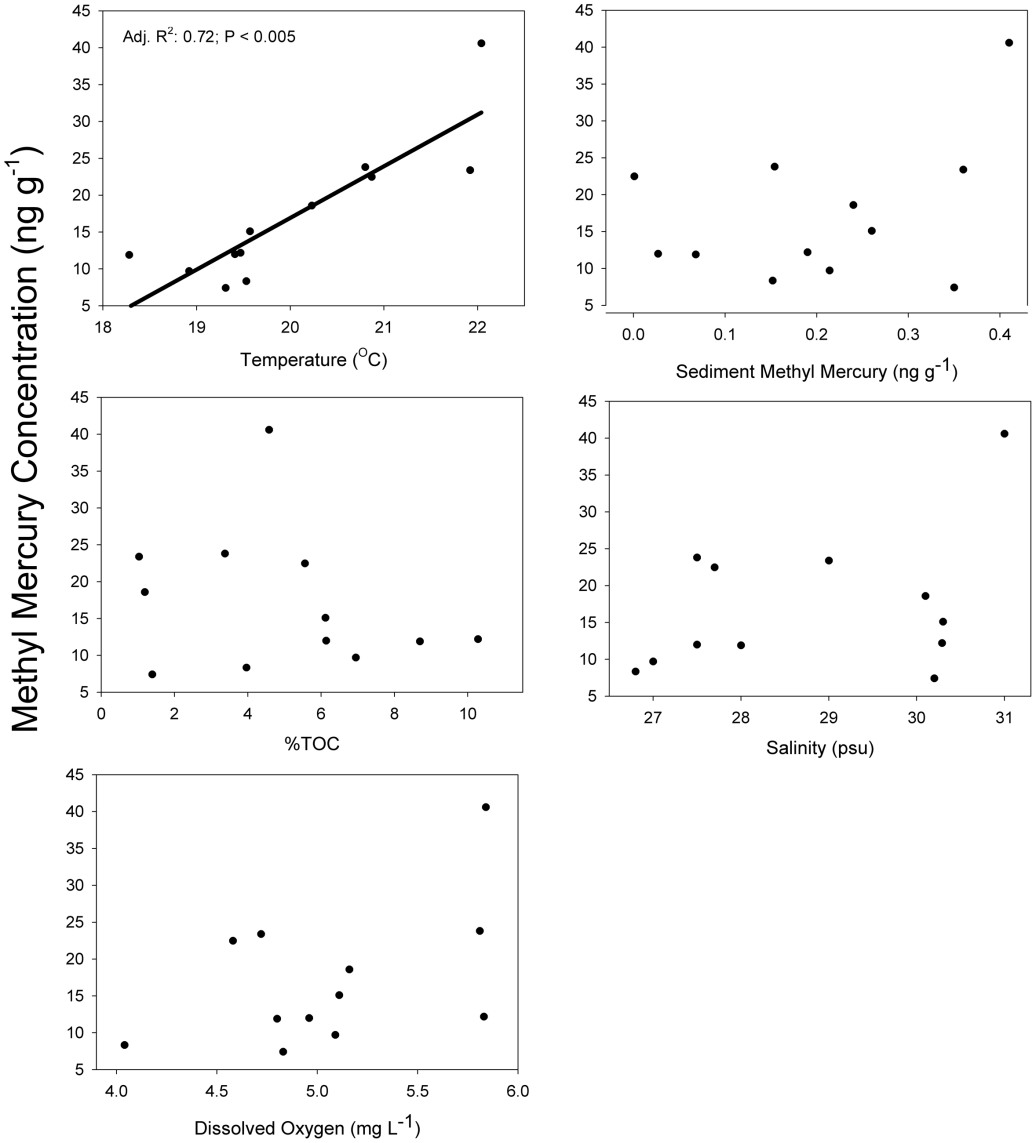
Mean tissue MeHg concentrations (ng g^−1^ dry fish tissue) in enclosed pools plotted against average individual environmental parameters collected from salt marsh pools. There are no significant relationships between fish MeHg concentrations and salinity, dissolved oxygen, %TOC, or sediment MeHg. In contrast to other environmental variables, temperature shows a significant positive regression with tissue MeHg concentrations.

**Figure 3:**
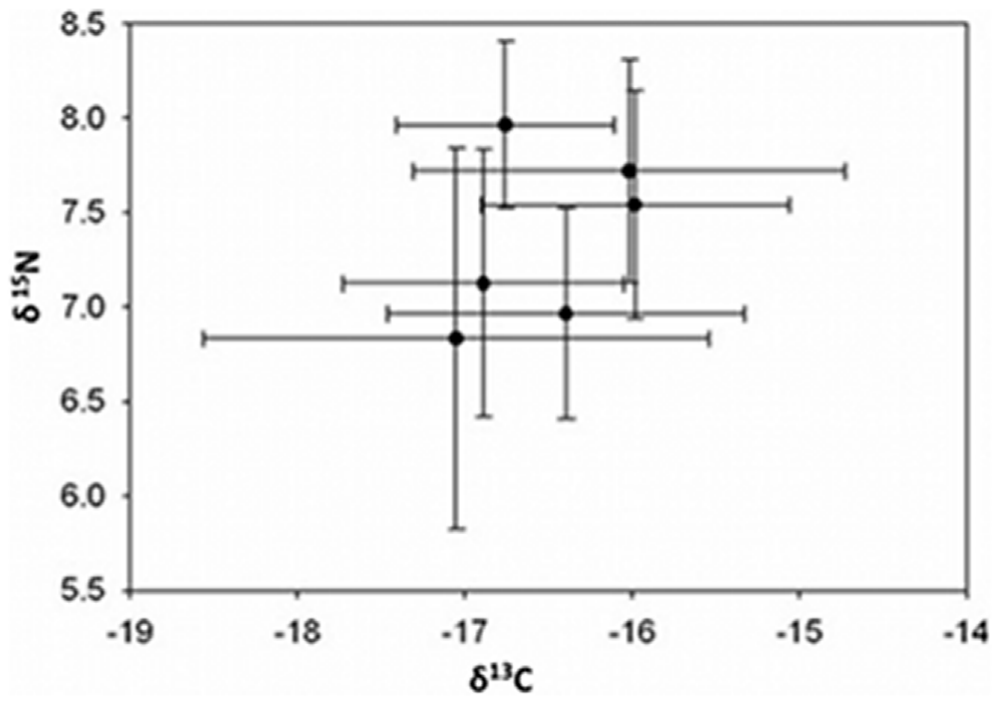
δ^13^C and δ^15^N stable isotopes values (±SD) for YOY killifish collected from natural pool habitats. Killifish collected from individual pools did not differ in either carbon or nitrogen isotopic signatures, suggesting that fish collected from different pools had similar diets and dietary composition did not affect MeHg bioaccumulation.

In the field studies, temperature was the only significant term in the multiple regression for MeHg concentrations with an adjusted r^2^ value of 0.72 (Table 1). There was no relationship between sediment %MeHg and temperature (MS = 1.89, F = 3.66, *P* = 0.088, adjusted r^2^ = 0.21; Fig. 1). Pools exhibited a range of values for selected environmental variables (Table 2). MeHg concentrations in sediments ranged from 0.001 to 0.214 ng g^−1^ MeHg in 2009 and 0.19 to 0.41 ng g^−1^ MeHg in 2010. Percent sediment TOC ranged from 3.96 to 8.70% in 2009 and 1.03 to 10.28% in 2010. Mean salinity, temperature and dissolved oxygen values in pools ranged from 26.8 to 28.0 PSU, 18.28 to 20.87°C, 4.04 to 5.81 mg/L in 2009 and 29.0 to 31.0 PSU, 19.31 to 22.04°C, 4.72 to 5.84 mg/L in 2010 (Fig. 2). No significant differences in stable isotope values of killifish were found among pools (Fig. 3), indicating that all fish were feeding on similar prey items.

**Table 1:** Multiple regression analysis for pool samples; temperature was the only significant predictor in the model.

Independent Variables	Regression Coefficients	SS	P-values	Adj. r^2^
Temperature (°C)	7.37	465.8	0.005	0.72
Dissolved Oxygen (mg L^−1^)	3.41	21.2	0.388	
% Total Organic Carbon	0.55	12.9	0.495	
Salinity (psu)	0.36	1.3	0.822	
Sediment MeHg (ng g^−1^)	−3.8	0.9	0.849	

**Table 2:** Environmental parameters, % TOC, methylmercury concentrations in sediment and tissue of *Fundulus heteroclitus* from salt marsh pools.

2009	%TOC	SedimentMeHg (ng g-1)	Mean Tissue MeHg (ng g-1)	Mean Temp. (°C)	Mean salinity (psu)	Mean DO (mg L^−1^)
Pool 1	3.96	0.15	8.34±2.96	19.53±0.01	26.8±0.87	4.04±0.47
Pool 2	6.14	0.03	14.18±1.31	19.41±0.12	17.5±1.12	4.96±0.47
Pool 3	8.70	0.07	9.12±7.04	18.28±0.08	28.0±0.89	4.80±0.47
Pool 4	5.56	0.001	22.48±5.76	20.87±0.13	27.7±0.99	4.58±0.27
Pool 5	6.94	0.21	9.07±2.94	18.92±0.11	27.0±1.03	5.09±0.58
Pool 6	3.38	0.15	23.81±11.05	20.80±0.08	27.5±0.99	5.81±0.63

2010						
Pool 1	1.39	0.35	7.42±2.36	19.31±0.06	30.3±0.72	4.83±0.95
Pool 2	1.19	0.24	18.6±4.34	20.23±0.07	30.1±1.12	5.16±0.65
Pool 3	10.28	0.19	12.2±3.09	19.47±0.08	30.3±1.05	5.83±0.76
Pool 4	1.03	0.36	23.4±7.42	21.92±0.08	29.0±1.31	4.72±0.68
Pool 5	6.12	0.26	15.1±2.35	19.57±0.06	30.3±0.33	5.11±0.80
Pool 6	4.58	0.41	40.59±5.28	22.04±0.09	31.0±0.71	5.84±0.56

**Figure 4:**
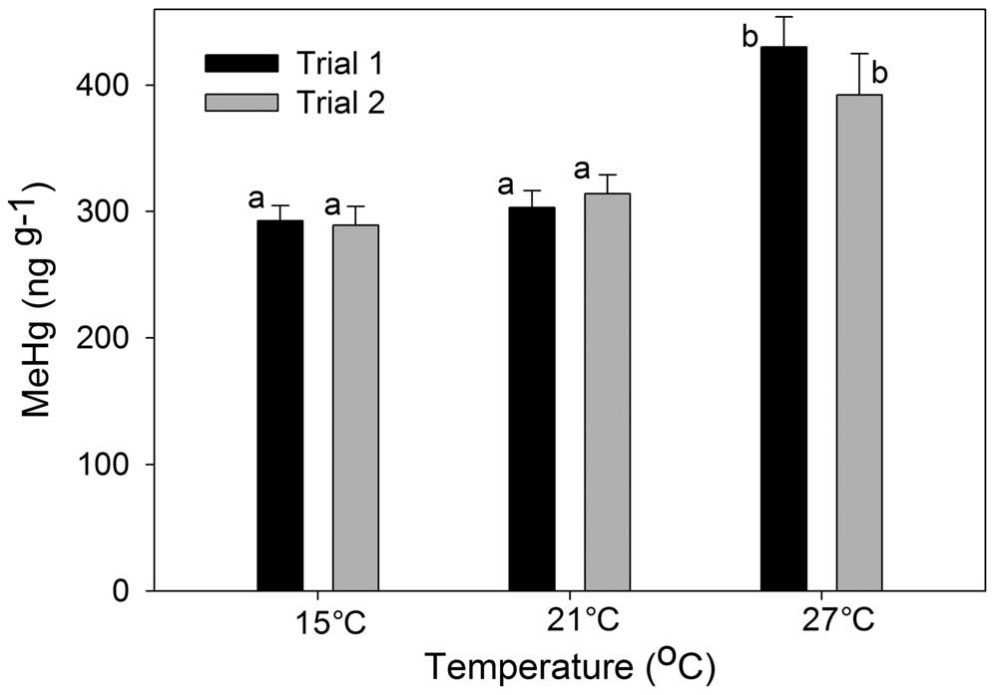
Mean± s.d. of MeHg concentrations of killifish (ng g^−1^ dry weight) exposed to 15°C, 21°C and 27°C for 30 days. MeHg concentrations were highest in killifish exposed to 27°C. Different letters indicate significant differences.

**Figure 5:**
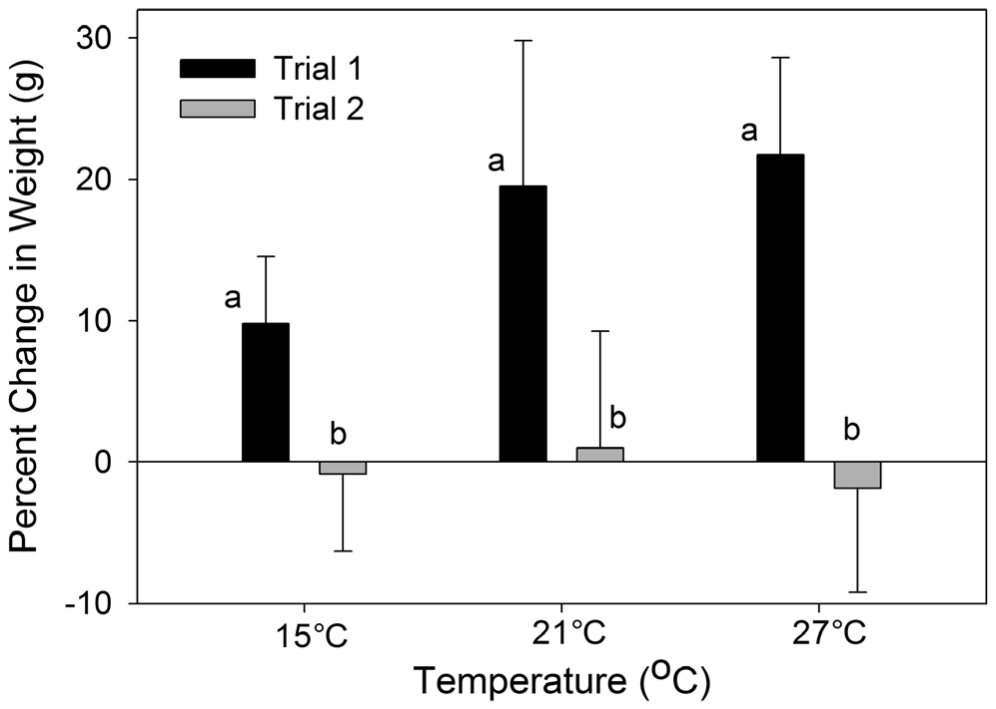
Mean± s.d. of growth (expressed as % weight change) of killifish exposed to 15°C, 21°C and 27°C for 30 days. Trial 1 killifish had positive growth in all treatment temperatures. Trial 2 killifish had slight negative average growth in treatment temperatures of 15°C and 27°C.

In laboratory experiments, we found a significant main effect of temperature on bioaccumulation of MeHg (Mean Squares (MS) = 0.02702, F2,2 = 10.13, P<0.001; Fig. 4) with greater concentrations of MeHg in fish exposed to the highest temperature, 27°C (P<0.05, Tukey-Kramer). No significant effects of trial or interaction between trial and temperature were observed (Sum of Squares (SS) = 0.0007, F1,2 = 0.269, P = 0.610; SS = 0.0040, F2,2 = 0.743, P = 0.487). Percent change in weight for each treatment was significantly different between trials, with slow or no growth in trial 2 (MS = 1927.8, F1,2 = 27.7, P<0.001; Fig. 5). No effect of trial or interaction of trial and temperature were found (SS = 299.5, F2,2 = 2.99, P = 0.070; SS = 295.9, F2 = 2.96, P = 0.072). One-way ANOVA revealed a significant difference in the initial sizes of killifish for the two trials (MS = 1.94, F1,2 = 19.24, P<0.001) with smaller initial sizes of trial 1 fish (0.921 g±0.075) than trial 2 fish (1.439 g±0.091).

### Bioenergetics Model

The proportion of maximum consumption (P) needed to produce the observed weight change during the 30 day laboratory averaged 19±2% with an estimated consumption that varied between 0.018 g g^−1^d^−1^to 0.048 g g^−1^d^−1^. MeHg assimilation efficiency (AE) from modeled consumption and fitted P values were similar across temperatures (mean values of 56%, 51% and 70% at 15°C, 21°C, 27°C). Based upon observed growth rates, the model indicated that temperature increased consumption in fish from both trials, but respiration also increased. Thus, assimilated MeHg concentrations were a function of low net relative growth of new tissue at warmer temperatures. The model indicates a possible metabolic mechanism for increased bioaccumulation: fish at the highest temperature ate more food (and thus ingested more MeHg) but grew less, leading to an increase in MeHg relative to fish at lower temperatures.

## Discussion

Our field and laboratory results indicate that warmer temperatures were linked to greater bioaccumulation of MeHg in the resident estuarine killifish, *Fundulus heteroclitus*. Killifish have a broad geographic range and are important prey for predatory fish that move into estuaries to feed and are harvested for human consumption [49], [61]. *F*. *heteroclitus* spend their entire lives in a single estuarine environment and accumulate MeHg through dietary uptake of zooplankton and small benthic invertebrates [62]–[64]. Across marine food webs, concentrations of MeHg that enter at the base of food webs will be propagated to higher trophic level species [33]. Thus, climate-induced increases in lower trophic level bioaccumulation will likely increase human exposure to MeHg.

Effects of temperature on MeHg bioaccumulation in killifish can follow from changes in fish metabolism, prey species composition, environmental conditions influencing sediment biogeochemistry, or MeHg production and bioavailability [23], [37]. Killifish consume a wide range of benthic and pelagic prey including detritus, insects, amphipods and copepods [65], [66]. Our field study indicates fish collected from different pools had similar diets, inferred from stable isotope values, suggesting that prey preference had little influence on MeHg bioaccumulation. The duration of exposure (i.e., field fish were exposed for multiple months as opposed to 30 days in the lab) to Hg did not qualitatively change the relationship of higher temperature and increased MeHg bioaccumulation. Therefore, the observed differences in magnitude of MeHg accumulation between field and laboratory experiments can be better explained by the consumption of MeHg enriched food by laboratory fish relative to natural levels in the field.

Warmer temperatures can influence both the physiology and metabolism of fish. Fish experiencing warmer temperatures typically have elevated metabolic rates, but consumption rates may increase or decrease depending on the changes in behavior and prey availability. The effect of temperature on MeHg accumulation depends largely on growth efficiency, which integrates the effects of consumption and metabolism [18], [44]–[46]. The bioenergetics model based on our laboratory results suggested that killifish consumption increased with temperature, but respiration increased even more, leading to low growth, low growth efficiency, and greater MeHg bioaccumulation. The model also predicted that killifish tissue growth is faster at lower temperatures due to reduced respiration rates. Faster growing killifish should have reduced MeHg concentrations than slow-growing fish due to somatic growth dilution [18], [67]. Given this, one might have expected the 27°C treatment from trial 2, which exhibited no growth, to have slightly elevated MeHg tissue concentrations than their trial 1 counterpart. This was not evident in the laboratory data and may be due to the duration of the experiment being too short for this difference to be evident in the fish (MeHg had not reached a steady state concentration). Alternatively, the highly MeHg enriched food source masked subtle effects of growth on bioaccumulation within a given temperature treatment. For natural populations, fish experiencing warmer temperatures during their growing season may have relatively increased body burdens of MeHg due to the effects of temperatures on their metabolism. However, this may be mediated or exacerbated by other environmental variables.

In our laboratory study, percent mass gain or loss in killifish differed between the two laboratory trials. Fish generally did not grow or lost mass in the second trial but not the first. The difference in growth between the trials was likely an allometric effect; average initial sizes were larger for killifish used in the second trial. Nonetheless, both trials resulted in significantly greater average MeHg concentrations in killifish exposed to the highest temperature, suggesting that the effects of temperature on growth efficiency were consistent across trials despite the overall difference in growth. There was no difference in MeHg concentrations between the low and middle temperature treatment in either trial, contrasting with the apparently linear effect of temperature in the field experiment. This difference may be due to the fact that fish had been housed at 21°C since their fall collection prior to the experiment; thus, only fish in the highest temperature treatment experienced an overall increase in temperature. We hypothesize that had the initial cohort been housed for long-term care at 15°C, both the 21°C and 27°C treatments would have indicated a significant increase in MeHg. Optimal growth for killifish that reside in the Western North Atlantic occurs at temperatures ranging from 24°C to 29°C and lethal temperature is above 36°C.

In addition to affecting fish physiology, increased temperatures could also alter environmental conditions that would change the bioavailability of MeHg to fish through changes in sediment biogeochemistry [24], [58]. In general, the relationship of temperature to MeHg production and flux are poorly understood for marine ecosystems [11], [42], [43]. Past studies indicate that elevated temperatures in mesocosms and in the field could result in sulfate reduction rates, which could lead to higher methylation rates and greater flux to the water column [68]–[70]. Though microbial activity was not explicitly examined in this study, there was no relationship between sediment MeHg and temperature. Past studies have used sediment MeHg/Hg_t_ ratio (%MeHg) as a measure of methylation efficiency [71], [72]. Field data indicated that sediment MeHg concentrations as well as %MeHg differed slightly between pools, but these differences were not related to temperature (Fig. 1) or to fish MeHg tissue (Fig. 2). In this study, MeHg water column concentrations were not measured; while it can be inferred from the lack of relationship between temperature and sediment %MeHg that temperature had no effect on methylation rates and MeHg bioavailability to fish, it is not shown empirically in this study.

Field data in this study also indicate that MeHg bioaccumulation did not statistically relate to salinity, TOC, or dissolved oxygen, all of which can affect metal bioavailability. However, between-year variation of MeHg bioaccumulation in the field results may have been caused by temporal variation in microbial activity of sulfate-reducing bacteria, or changes to the prey community structure, salinity and ecosystem productivity [13], [22]-[24], [27], [29], [73]. The relationship between salinity and MeHg accumulation is still unclear as studies report both a positive and a negative relationship with increasing salinity and are typically based on aqueous rather than dietary exposure [73], [74]. Our average difference between years (∼4 psu) would not have likely affected MeHg concentrations as a recent study demonstrated there was little difference in MeHg concentrations in killifish exposed to experimental salinities ranging from 0 to 25 psu [73]. The field results of this study suggest that across the range of environmental variables, MeHg concentration differences in killifish were only correlated with temperature (Fig. 2). The most parsimonious explanation for these results is provided by the bioenergetics model of our laboratory fish study that indicated that fish exposed to higher temperatures ate more and grew less than fish exposed to lower temperatures. This is consistent with other studies that indicate slow-growing fish have higher MeHg concentrations than fast-growing fish [18], [47].

Climate change encompasses multiple changes in physical factors (e.g., wind flows, atmospheric circulation, and precipitation) that will likely affect mercury deposition and bioavailability, and biological factors (e.g., species biogeographic patterns, composition) that affect food web structure and in turn bioaccumulation [30]. We have shown that under current predicted changes in climate warming, MeHg concentrations significantly increased under controlled laboratory and natural field mesocosm studies. However, other climate related factors would likely indirectly affect contaminant bioaccumulation in resident fish. Earlier estuarine ice-out dates and earlier winter/spring high river flows will result in longer ice-free seasons that can lengthen the growing season of killifish and affect its growth efficiency in addition to average summer temperatures. Moreover, changes in nutrient loading in estuaries due to changes in precipitation could also alter estuarine trophic status thereby changing MeHg production and bioaccumulation [16], [17], [75]. Nixon et al [34] have shown a reduction in overall productivity in Narragansett Bay with long term changes in climate and reduced productivity in freshwater systems has been shown to result in increases in MeHg bioaccumulation [29], [76].

While many of these factors have the potential to indirectly alter MeHg bioavailability to marine food webs, the predicted increases in temperature will directly increase MeHg bioaccumulation at lower trophic levels such as killifish. This increase can be propagated to higher trophic level fish consumed by humans, resulting in increased human exposure to MeHg. This effect should be incorporated into policy and management efforts aimed at reducing human health risks from MeHg exposure.
